# Impact of different anesthesia subspecialties on anxiety levels and sleep quality among anesthesiologists: a cross-sectional study

**DOI:** 10.3389/fpubh.2025.1733167

**Published:** 2025-12-15

**Authors:** Bo Song, Zhu Wen, Ying-hao Guo, Hong-xia He, Kun Peng, Jun Li

**Affiliations:** Mianyang Key Laboratory of Anesthesia and Neuroregulation, Department of Anesthesiology, Mianyang Central Hospital, Mianyang, Sichuan, China

**Keywords:** anesthesiologists, occupational stress, anxiety, sleep quality, subspecialty

## Abstract

**Background:**

Anesthesiologists face high risks of occupational burnout, anxiety, and sleep disorders. Significant subspecialization within the field suggests different specialties may constitute distinct stressors, but whether this leads to systematic variations in mental health outcomes remains unclear.

**Objective:**

This study aimed to investigate the association between different anesthesia subspecialties and the levels of anxiety, depression, and sleep quality among anesthesiologists.

**Methods:**

A multi-center cross-sectional study was conducted. Eighty-five anesthesiologists from four tertiary Grade A general hospitals completed the Generalized Anxiety Disorder-7 (GAD-7), Patient Health Questionnaire-9 (PHQ-9), and Pittsburgh Sleep Quality Index (PSQI) questionnaires. Retrospective work data were extracted from departmental systems. Statistical analyses included one-way ANOVA, Tukey's Honest Significant Difference (HSD) *post-hoc* tests, and multiple linear regression controlling for key covariates.

**Results:**

Significant differences were found in GAD-7 (*F* = 4.32, *p* < 0.01), PHQ-9 (*F* = 4.98, *p* < 0.001), and PSQI (*F* = 5.18, *p* < 0.001) scores across subspecialties. Multiple linear regression, adjusting for weekly overtime, monthly night shifts, age, experience, and gender, confirmed that the primary subspecialty was independently associated with anxiety (β = 0.35, *p* = 0.003), depression (β = 0.38, *p* = 0.001), and poor sleep quality (β = 0.41, *p* < 0.001).

**Conclusion:**

The anesthesia subspecialties in which an anesthesiologist works is independently associated with the risk of anxiety, depression, and sleep disturbances. These findings suggest that targeted support strategies should be considered for anesthesiologists in high-stress subspecialties such as cardiothoracic and pediatric anesthesia.

## 1 Introduction

Anesthesiology serves as a critical pillar supporting the advancement of modern surgery, characterized by its high technical demands, significant uncertainty, and substantial responsibility. In China, a workforce of approximately 60,000 anesthesiologists and anesthesia residents, alongside about 7,000 anesthesiologist assistants, supports the healthcare system, with non-physician anesthesia providers being relatively scarce. Within this context, overtime work is a common occurrence (1). Substantial evidence indicates that anesthesiologists represent a high-risk group for occupational burnout, anxiety, and depression (2–4). In China, the pronounced disparity between limited anesthesia human resources and extensive clinical demand makes overtime work routine. Sleep deprivation and fatigue have been demonstrated to significantly increase the risk of medical errors, seriously jeopardizing both patient safety and physician health (5–7). However, previous studies have predominantly treated anesthesiologists as a homogeneous group in their assessments (4).

Despite considerable subspecialization within anesthesiology departments, where distinct surgical specialties—such as the extreme precision and prolonged duration of cardiac surgery, the unpredictability of obstetric and pediatric cases, and the time-sensitive decision-making required in emergency settings—constitute differentiated stressors (8, 9), systematic investigation into the association between such specific stress exposures and precise mental health indicators like anxiety, depression, and sleep quality remains insufficient. Although preliminary research (10) has revealed variations in burnout levels across subspecialties, a critical knowledge gap persists: does the qualitative nature of work stress exert specific effects on anesthesiologists' mental health, independent of quantitative workload measures?

Therefore, this study aimed to investigate the associations between primary assigned surgical subspecialty and the levels of anxiety, depression, and sleep quality among anesthesiologists. We hypothesized that working in high-stress subspecialties would be independently associated with worse mental health outcomes, even after adjusting for quantitative workload measures.

## 2 Methods

### 2.1 Study design and ethical approval

This multi-center cross-sectional study was approved by the Biomedical ethics committee of Mianyang (Approval No: S202503189-01). Written informed consent was obtained from all participants prior to the survey.

### 2.2 Study participants and sample size

The study enrolled anesthesiologists who had been continuously working in the anesthesiology departments of four tertiary Grade A general hospitals in our city between January 1, 2020, and December 31, 2024. A cluster sampling method was employed to invite all eligible anesthesiologists from these centers.

Inclusion criteria:

(1) Full-time anesthesiologists with ≥5 years of continuous service during the specified period;
(2) Competency in performing anesthesia across all major anesthesia subspecialties.

Exclusion criteria:

(1) Cumulative leave (e.g., maternity/sick leave) exceeding 6 months during the study period;
(2) Pre-existing diagnosis of psychiatric disorders prior to the study;
(3) Declined to participate in the questionnaire survey.

The sample size was estimated using G^*^Power 3.1 software. With an effect size *f* = 0.35, α error probability = 0.05, and statistical power (1–β) = 0.80 for one-way ANOVA, the required total sample size was approximately 84. A total of 85 valid questionnaires were ultimately collected, meeting the estimated requirement.

### 2.3 Data collection

Data collection comprised two components: retrospective work data extraction and prospective questionnaire administration.

#### 2.3.1 Retrospective work data extraction

The following anonymized data were extracted from the hospital human resource information systems and anesthesia department scheduling systems of participating hospitals:

- Demographic data: age, gender, years of professional experience, and professional title.
- Work exposure data:
  ° Primary surgical subspecialty: the cumulative months worked in predefined subspecialties over the past 5 years were calculated. The subspecialties were defined as: Cardiothoracic, Neurosurgical, Pediatric, Emergency (obstetric), General Surgery, and Ambulatory Surgery anesthesia. The subspecialty with the longest cumulative duration was designated as the “primary work subspecialty.”
  ° Workload: total overtime hours and total night shifts over the past five years were extracted, from which the weekly average overtime (hours/week) and monthly average night shifts (times/month) were computed.

#### 2.3.2 Questionnaire instruments

In October 2025, all eligible participants were surveyed using a unified electronic questionnaire, which included the following validated scales:

- Generalized Anxiety Disorder-7 (GAD-7) (12): assessed the severity of anxiety symptoms over the past 2 weeks. This 7-item scale yields a total score ranging from 0 to 21, with higher scores indicating more severe anxiety.
- Patient Health Questionnaire-9 (PHQ-9) (13): evaluated the severity of depressive symptoms over the past 2 weeks. This 9-item instrument provides a total score between 0 and 27, where higher scores reflect more severe depression.
- Pittsburgh Sleep Quality Index (PSQI) (3): measured subjective sleep quality over the past month. The 19 self-rated items generate seven component scores and a global score ranging from 0 to 21; a global score >7 typically indicates poor sleep quality.

### 2.4 Data matching and confidentiality

A unique coding system was used to link retrospectively extracted work data with questionnaire responses while protecting participant privacy. All analyses were conducted in a fully de-identified environment. Original data were accessible only to research team members and stored on encrypted servers.

### 2.5 Statistical analysis

All analyses were performed using SPSS Statistics (Version 26.0; IBM Corp., USA). A two-sided *p*-value <0.05 was considered statistically significant.

Descriptive statistics: continuous variables following a normal distribution are presented as mean ± standard deviation (SD); categorical data are expressed as counts (percentages).

Group comparisons: one-way ANOVA was used for continuous variables, and the Chi-square test for categorical variables across groups.

*Post-hoc* multiple comparisons were conducted using Tukey's Honest Significant Difference (HSD) test to conservatively control the family-wise error rate during multiple pairwise testing. Three separate multiple linear regression models were constructed with GAD-7, PHQ-9, and PSQI scores as dependent variables to adjust for potential confounding effects of weekly overtime hours, monthly night shifts, age, years of experience, and gender. Results are reported as standardized regression coefficients (β) with their standard errors (SE).

## 3 Results

### 3.1 Participant flow and response rate

A total of 105 questionnaires were distributed, with 85 valid responses returned, yielding a valid response rate of 81%.

### 3.2 Baseline characteristics and workload of study participants

Participants were categorized into six groups according to their primary surgical subspecialty. As shown in Table 1, no significant differences were observed among the groups in terms of age, gender, years of experience, or professional title distribution (*p* > 0.05). However, workload indicators differed significantly across groups (*p* < 0.001). Specifically, the cardiothoracic anesthesia, pediatric anesthesia, and emergency anesthesia groups demonstrated significantly higher weekly overtime hours and monthly night-shift frequencies compared to other groups, whereas the ambulatory surgery anesthesia group reported the lightest workload.

**Table 1:** Comparison of baseline characteristics and workload among anesthesiologists by subspecialty group (*n* = 85).

**Variable**	**Total (*n* = 85)**	**Cardiothoracic (*n* = 12)**	**Neurosurgica (*n* = 10)**	**Pediatric (*n* = 15)**	**Emergency (*n* = 13)**	**General surger (*n* = 20)**	**Ambulator (*n* = 15)**	**Statistic**	***p*-Value**
Age (years)	36.5 ± 8.2	38.2 ± 7.5	40.1 ± 6.8	34.8 ± 5.2	35.3 ± 9.1	37.1 ± 8.5	35.0 ± 7.2	*F* = 0.92	0.481
Gender (Male/Female)	48/37	8/4	7/3	5/10	9/4	14/6	5/10	χ^2^=9.15, df = 5	0.104
Experience (years)	10.3 ± 7.5	12.1 ± 6.8	14.5 ± 5.9	8.2 ± 4.1	9.8 ± 8.2	10.9 ± 7.8	7.5 ± 6.0	*F* = 1.87	0.093
**Professional title [*****n*** **(%)]**								
Resident	25 (29.4)	3 (25.0)	2 (20.0)	6 (40.0)	4 (30.8)	6 (30.0)	4 (26.7)		
Attending	35 (41.2)	5 (41.7)	4 (40.0)	6 (40.0)	5 (38.5)	9 (45.0)	6 (40.0)	χ^2^ = 4.82, df = 10	0.567
Associate Chief or above	25 (29.4)	4 (33.3)	4 (40.0)	3 (20.0)	4 (30.8)	5 (25.0)	5 (33.3)		
Weekly Overtime (hours)	26.4 ± 9.1	31.5 ± 3.1	28.0 ± 4.5	35.2 ± 2.8	32.8 ± 3.5	22.1 ± 3.8	13.5 ± 2.9	*F* = 55.32, df = 6,78	<0.001
Monthly Night (Shifts)	3.0 ± 1.1	3.4 ± 0.5	3.1 ± 0.7	4.1 ± 0.6	3.7 ± 0.8	2.6 ± 0.7	1.5 ± 0.5	*F* = 45.17, df = 6,78	<0.001

Data are presented as mean ± standard deviation or count (percentage). One-way ANOVA was used for continuous variables and Chi-square test for categorical variables. The unequal distribution of participants across subspecialty groups reflects the natural clinical workforce distribution. The total sample size of 85 was determined to be adequate by a priori power analysis (power = 0.80, α = 0.05, effect size f = 0.35).

### 3.3 Comparison of anxiety, depression, and sleep quality scores across subspecialty groups

One-way ANOVA revealed statistically significant differences in GAD-7 anxiety scores (*F* = 4.32, *p* < 0.01), PHQ-9 depression scores (*F* = 4.98, *p* < 0.001), and PSQI sleep quality scores (*F* = 5.18, *p* < 0.001) among the different subspecialty groups, as detailed in Table 2.

**Table 2:** Comparison of GAD-7, PHQ-9, and PSQI scores across subspecialty groups (Mean ± SD).

**Group**	***n***	**GAD-7 Score**	**PHQ-9 Score**	**PSQI Score**
Cardiothoracic	12	10.2 ± 3.1^c^	12.8 ± 3.3 ^d^	12.5 ± 2.8 c
Neurosurgical	10	8.5 ± 2.8 ^b^	9.5 ± 2.9^b^	10.1 ± 2.5^b^
Pediatric	15	9.8 ± 2.9^b, c^	11.2 ± 3.0 ^c^	11.8 ± 2.5 c
Emergency	13	9.2 ± 3.0 ^b, c^	10.1 ± 2.8 ^b, c^	10.9 ± 2.6^b, c^
General Surgery	20	6.0 ± 2.5^a^	7.2 ± 2.4 ^a^	7.5 ± 2.0 ^a^
Ambulatory	15	5.1 ± 2.2^a^	5.8 ± 2.1 ^a^	6.2 ± 1.9^a^
*F*-value		4.32	4.98	5.18
*p*-Value		<0.01	<0.001	<0.001

Values are presented as mean ± standard deviation. Means in the same column that do not share a common superscript letter (a, b, c, d) are significantly different based on Tukey's HSD *post-hoc* test (p <0.05).

### 3.4 Multiple linear regression analysis of factors influencing anxiety, depression, and sleep quality

**Figure 1:**
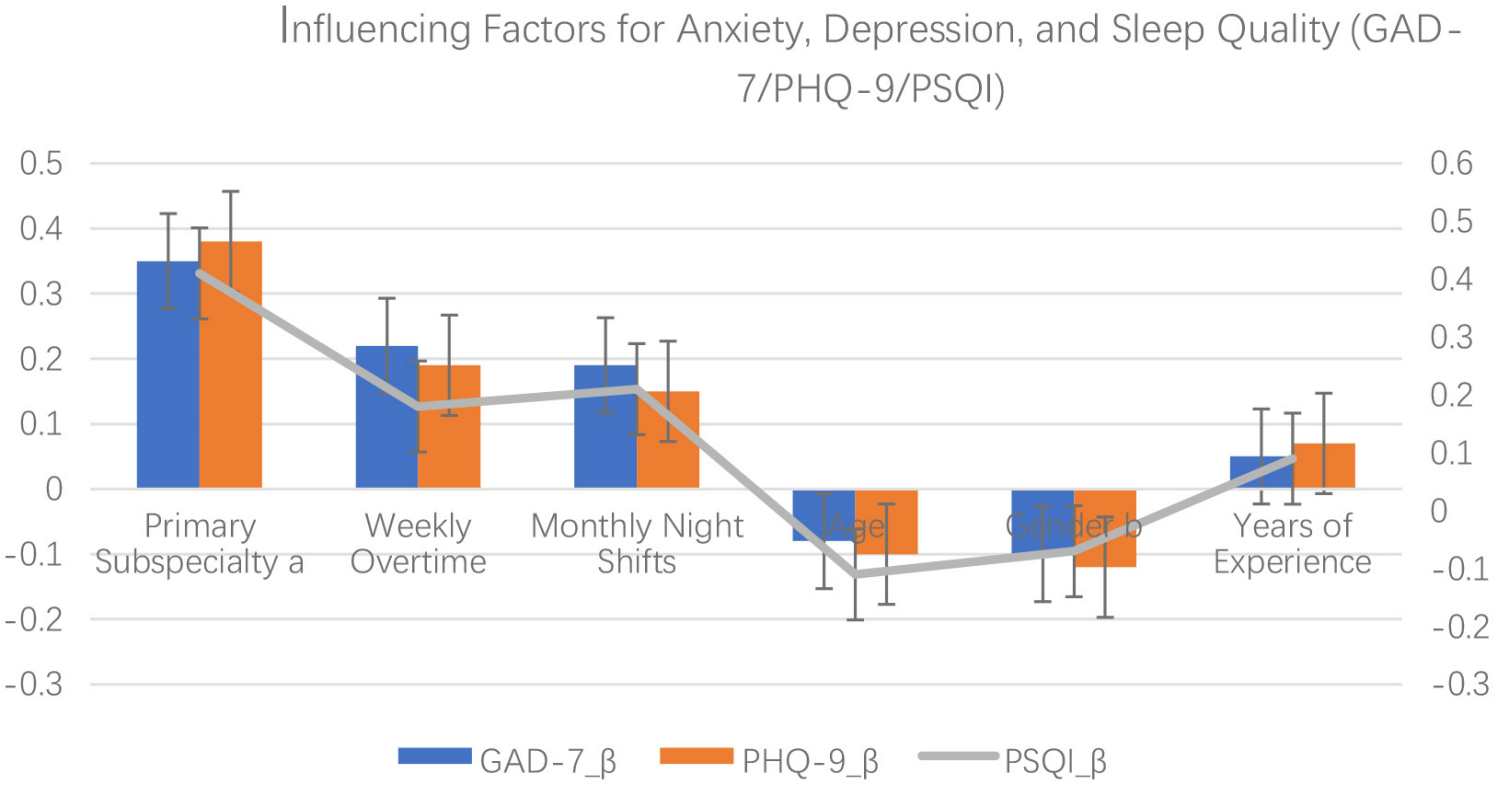
Multiple linear regression analysis of factors influencing anxiety (GAD-7), depression (PHQ-9), and sleep quality.

Multiple linear regression was performed to control for potential confounding factors. As presented in Table 3 and Figure 1, after adjusting for weekly overtime, monthly night shifts, age, years of experience, and gender, the primary work subspecialty (treated as an ordinal variable) was identified as an independent positive predictor of GAD-7 anxiety scores (β = 0.35, *p* = 0.003), PHQ-9 depression scores (β = 0.38, *p* = 0.001), and PSQI sleep quality scores (β = 0.41, *p* < 0.001). Weekly overtime hours and monthly night shifts did not show independent significant effects in any of the models (*p* > 0.05).

**Table 3:** Multiple linear regression analysis of factors influencing anxiety (GAD-7), depression (PHQ-9), and sleep quality (PSQI).

**Variable**	**GAD-7 Score**		**PHQ-9 Score**		**PSQI Score**	
	β **(SE)**	***p*** **-Value**	β **(SE)**	***p*** **-Value**	β **(SE)**	***p*** **-Value**
Primary Subspecialty^a^	0.35 (0.11)	0.003	0.38 (0.12)	0.001	0.41 (0.10)	<0.001
Weekly Overtime (hours)	0.22 (0.12)	0.061	0.19 (0.13)	0.135	0.18 (0.11)	0.12
Monthly night shifts (times)	0.19 (0.11)	0.088	0.15 (0.12)	0.205	0.21 (0.10)	0.052
Age (years)	−0.08 (0.10)	0.452	−0.10 (0.11)	0.378	−0.11 (0.09)	0.301
Gender^b^	−0.10 (0.10)	0.321	−0.12 (0.11)	0.275	−0.07 (0.09)	0.495
Years of experience	0.05 (0.11)	0.662	0.07 (0.12)	0.559	0.09 (0.10)	0.412
Model R^2^	0.28		0.31		0.33	
Model *p*-Value	<0.001		<0.001		<0.001	

a: “Primary work subspecialty” was treated as an ordinal variable based on presumed stress levels, assigned values as follows: 1 = Ambulatory surgery, 2 = General surgery, 3 = Neurosurgical, 4 = Emergency, 5 = Pediatric, 6 = Cardiothoracic.

^b^Gender assignment: Male = 1, Female = 2.

SE, Standard Error.

## 4 Discussion

This multi-center cross-sectional study systematically evaluated the association between working across different anesthesia subspecialties and the mental health of anesthesiologists. The key finding indicates that the specific surgical subspecialty in which an anesthesiologist works is independently associated with anxiety, depressive symptoms, and sleep quality—a relationship that persists after adjustment for conventional workload indicators such as overtime hours and night-shift frequency. Compared to low-stress subspecialties like ambulatory surgery, anesthesiologists in high-pressure environments such as cardiothoracic and pediatric surgery showed significantly elevated scores across all three dimensions. This pattern aligns with findings reported by Yang et al. (10) regarding the co-occurrence of occupational stress and mental health issues among anesthesiologists.

Our results align with the report by Sanfilippo et al. (11), which indicated higher burnout rates among cardiac anesthesiologists. However, our study extends these findings by quantifying this risk and broadening its scope to encompass multiple dimensions, including anxiety, depression, and sleep quality. More importantly, the multiple regression analysis revealed a key insight: the qualitative nature of work stress, inherent to the subspecialty's characteristics, may exert a more fundamental impact on psychological wellbeing than the quantitative aspects of workload. This finding resonates strongly with the Job Demands-Resources (JD-R) mode (6). High-stress subspecialties (e.g., cardiothoracic, pediatric) not only present extreme job demands—such as life-or-death decision-making, high emotional labor, and clinical unpredictability—but may also offer insufficient job resources (e.g., decision latitude, social support, adequate rewards) to buffer these demands, thereby leading to psychological depletion and health deterioration (6, 14). This pattern is highly consistent with the core principle of Siegrist's Effort-Reward Imbalance model (14).

Particularly noteworthy is that PHQ-9 depression scores demonstrated the strongest association (β = 0.38). This strongly suggests that chronic exposure to high-stress subspecialty environments may not only precipitate acute emotional distress and poor sleep but could also progress to more severe, clinically significant depressive states requiring intervention (15). This finding holds particular significance within the Chinese context, characterized by a substantial anesthesia workforce striving to meet immense clinical demands with limited resources (1). It corroborates conclusions from other domestic studies reporting high prevalence of depressive symptoms among physician populations (16, 17), while precisely localizing this risk to specific high-risk subspecialty groups. Extensive systematic reviews confirm that anesthesiologists' chronic exposure to high-intensity work pressure is a primary cause of their prevalent mental health issues (4), with this pressure being especially pronounced in high-risk subspecialties like cardiac anesthesia (18).

The findings of this study carry clear practical implications. They suggest that hospital and departmental managers must look beyond mere “working hours” on schedules when optimizing human resources and critically consider the “stress attributes” of work content. The management and organization of anesthesiologists and anesthetist nurses constitute an integrated system; strict enforcement of personnel access, supervision, and assessment systems is essential (19). Consequently, for anesthesiologists in high-stress subspecialties, targeted strategies should be considered. Future research and management policies could explore establishing scientific subspecialty rotation systems to prevent prolonged, sustained exposure to extreme stress; providing dedicated psychological support resources, such as tailored group counseling and peer support systems; and fostering psychological capital and professional resilience through proactive team building and recognition mechanisms (20, 21). Systematic reviews indicate that targeted interventions can effectively combat burnout among anesthesiologists (22). Furthermore, the generally poor sleep quality among physicians in high-stress subspecialties means that fatigue is not merely a personal health issue but also a serious safety hazard (7).

### 4.1 Limitations and future directions

This study has several limitations. First, the cross-sectional design precludes causal inference. Reverse causality remains possible, whereby anesthesiologists with poorer baseline psychological health might self-select or be assigned to lower-stress subspecialties. Second, our assessment relied primarily on subjective scales; future research could incorporate objective measures (e.g., actigraphy for sleep monitoring, salivary cortisol as a physiological stress marker) for triangulation. Although our results resonate with findings from other high-stress medical specialties in China, such as dentistry (23) and general practice (17). Third, To enhance the statistical rigor of our findings, all *post-hoc* comparisons were re-analyzed using Tukey's HSD test, which provides robust control for the Type I error rate. Finally, the sample was drawn from four tertiary hospitals, so the generalizability of the results requires cautious consideration.

Based on the findings and limitations of this study, future research could focus on the following areas: (1) conducting prospective cohort studies to establish the temporal sequence and causal relationship between subspecialty work exposure and mental health outcomes; (2) delving deeper into the specific mechanisms underlying the high stress in particular subspecialties (e.g., identifying the most critical job characteristics) to provide targets for precise interventions; (3) developing and evaluating the effectiveness of psychological intervention programs tailored to these high-risk groups; and (4) investigating the actual impact of burnout and depression on the work performance and patient safety of anesthesiologists in high-stress subspecialties.

## Data Availability

The raw data supporting the conclusions of this article will be made available by the authors, without undue reservation.
